# 

**Published:** 1868-10-01

**Authors:** 


### CHICAGO MEDICAL JOURNAL.

J. ADAMS ALLEN, M.D., LL.D., Editor. WALTER HAY, A.M., M.D., Associate Editor.

All letters for the JOURNAL should be addressed to Dr. J. ADAMS ALLEN, P. O. Box 1948, Chicago, Ill.

TERMS — $3 Per Annum, in Advance;

$4 if not paid before 1st of July, in each year. Single copies, 25 cents.

*CONTENTS:*

### *BOOKS RECEIVED.*

DISEASES OF CHILDREN: A Clinical Treatise Based on Lectures delivered at the Hospital for Sick Children, London. By Thomas Hillier, M.D., London, etc., etc. Philadelphia: Lindsay & Blakiston, 1868. Pp. 402. Chicago: W. B. Keen & Co. $3.00.

This book consists essentially in a series of short monographs on the diseases of children between two and twelve years of age. Surgical diseases are omitted. It is a very useful contribution to the literature of the subject. The American edition is afforded us in the usual elegant style of the Philadelphia house whose imprint it bears.

THE PHYSICIAN'S VISITING LIST FOR 1869: Eighteenth Year of its publication. Philadelphia: Lindsay & Blackiston. Sold by booksellers and druggists.

THE PHYSICIAN'S HAND-BOOK FOR 1869. By William Elmer, M.D. and Albert D. Elmer, M.D. New York: W. A. Townshend & Adams, Publishers, No. 434 Broome Street. 1869.

NEW MEDICAL JOURNAL. THE CALIFORNIA MEDICAL GAZETTE: A Monthly Journal of Medical and Surgical Science. Published at San Francisco, by A. Roman & Co.

A double column quarto comes to us in good typographical style, and with good paper. We regret that no editorial name appears, although its contents show it to have received good editorial supervision. $5.00 a year (gold) in advance. Single copies 50 cents. Roman friends, your type is too uniformly small. You will spoil your readers' eyes.

AITKIN'S SCIENCE AND PRACTICE OF MEDICINE. Second American from the Fifth London Edition. Now ready. In

2 Volumes, containing 2,000 Royal Octavo Pages, a Colored Map, a Lithographic Plate, and One Hundred and Thirty Illustrations on Wood. The Science and Practice of Medicine. By William Aitken, M.D., Professor of Pathology in the Army Medical School, etc., etc. Second American from the Fifth Enlarged and Carefully Revised London Edition, with Large Additions by Meredith Clymer, M.D., Ex-Professor of the Institutes and Practice of Medicine in the University of New York; formerly Physician to the Philadelphia Hospital, etc. In 2 Volumes Royal Octavo. Price, bound in Cloth, beveled boards, $12.00; Leather, $14.00. W. B. Keen & Co., Chicago.

Fifteen months have been spent by Dr. Aitken in thoroughly revising this *Great Work*, and adding to it many valuable additions and improvements, amounting to about 100 pages of new matter, included in which will be found the adoption and incorporation in the text of the "*new nomenclature of the Royal College of Physicians of London;*" to which are added the *Definitions* and the foreign equivalents for their English names.

The subjects of *Malignant Cholera*, of *Paralysis*, of *Epidemic Cerebro-Spinal Meningitis*, and of *Intestinal Obstruction* have been entirely re-written; and several other subjects in connection with the treatment of disease, of the greatest importance, are considered for the first time in this edition.

The first American edition of this work was out of print in little more than twelve months after publication. So rapid a sale may be accepted as an evidence of its appreciation by the profession of this country, and as a recognition of its claim to being a fair exposition of the Medical Science and Art of the day.

In the present edition the editor has carefully revised his contributions, and added much new material. His additions are equal to about three hundred pages of the London edition. They will be chiefly found under the heads of: *Lardaceous Degeneration*, *Vaccination*, *Measles*, *Erysipelas*, *Typhoid*, *Relapsing*, *Yellow and Malarial Fevers*, *Dysentery*, *Malignant Cholera*, *Malignant Pustule*, *Syphilis*, *Pathology of the Dietic Diseases*, *Scurvy*, *Parasitic Diseases*, *Rheumatism*, *Gout*, *Chronic Bright's Disease*, *Cancer*, *Tuberculosis*, *Diseases of the Nervous System*, *Diseases of the Heart and Lungs*, *the Sphygmograph Pyæmia*, *Diseases of the Digestive Organs*, *Diseases of the Kidneys*, and *Diseases of the Cutaneous System*.

They also including *twenty-two new articles* upon subjects not treated of, or only incidentally mentioned, by the Author, namely:

Camp Measles.

Spinal Symptoms in Typhoid Fever.

Typho-Malarial Fever.

Chronic Malarial Toxæmia.

Chronic Camp Dysentery.

Cholera Morbus.

Cholera Infantum.

Hereditary Syphilis.

Gonorrhœal Rheumatism.

Corpulence.

Physical Diagnosis of the Diseases of the Brain and Spinal Chord.

Delirium of Inanition.

Chronic Alcoholism.

Epileptiform Neuralgia.

Auscultation in Health and Disease.

Capillary Bronchitis.

Plastic Bronchitis.

Dilatation of the Bronchia.

Fibroid Degeneration of the Lung.

The Inoculation of Tubercle.

Chronic Pyæmia.

Syphilitic Disease of the Liver.

The subjects of Locomotor Ataxy, Glosso-Pharyngeal Paralysis, Aphasia, Dilatation of the Bronchia, the Sphygmograph and its tracings in disease, were introduced into this text-book by the Editor in the first American Edition (1866). They were first treated of by the Author in the Fifth English Edition (1868), and his articles on these disorders are chiefly condensed from those of the Editor, with the exception of the one on Dilatation of the Bronchia, which Dr. Aitkin has abridged from Dr. T. G. Stewart's excellent article in the Edinburgh Medical and Surgical Journal, December, 1867.

The issue of the first edition of this magnificent work was duly credited in the JOURNAL. At present we have only space to reiterate our warm commendation of the work as the leading one of its department. No physician who has the slightest claim to keeping pace with Medical Science, as it is understood in the present age, can afford to forego the possession of this (we were about to write *encyclopædia*) complete, reliable, common sense and practical treatise on the Practice of Medicine. Hereafter, when the pressure on our pages will permit, we shall take occasion to call attention to many particulars wherein Aitken's Practice excels all previously published books of reference on the subject.

